# A two-variable model robust to pacemaker behaviour for the dynamics of the cardiac action potential

**DOI:** 10.1016/j.mbs.2016.08.010

**Published:** 2016-11

**Authors:** Cesare Corrado, Steven A. Niederer

**Affiliations:** Division of Imaging Sciences & Biomedical Engineering, King’s College London, London SE17EH, United Kingdom

**Keywords:** Mitchell–Schaeffer model, Pacemaker behaviour, Parameter fitting

## Abstract

Ionic models with two state variables are routinely used in patient specific electro-physiology simulations due to the small number of parameters to be constrained and their computational tractability. Among these models, the Mitchell and Schaeffer (MS) action potential model is often used in ventricle electro-physiology due to its ability to reproduce the shape of the action potential and its restitution properties. However, for some choices of parameters characterising this ionic model, unwanted pacemaker behaviour is present. The absence of any a priori criterion to exclude unstable parameter combinations affects parameter fitting algorithms, as unphysiological solutions can only be discarded a posteriori. In this paper we propose an adaptation of the MS model that does not exhibit pacemaker behaviour for any combination of the parameters. The robustness to pacemaker behaviour makes this model suitable for inverse problem applications.

## Highlights

• Simplified cardiomyocyte electrophysiology model optimised for patient specific modelling.
• Modified Mitchell and Schaeffer model.
• No spurious pacemaker behaviour.
• Suitable for parameter fitting.
• Consistent leading order APD approximation.

## 1 Introduction

Cardiac ionic models are mathematical models describing the electrical response of a cardiac myocyte following an applied electrical stimulus.

When an electrical stimulus is applied to a cardiac myocyte, an action potential is generated by the flux of ionic species across the cell membrane. Complex mathematical models [2], [15], [24] describe the ionic current generated by each ionic species. Though physiologically accurate, these models are expensive to solve numerically due to their large number of state variables and their non-linear formulation; moreover, when personalising such models, an additional challenge arises from the large number of parameters to constrain.

In contrast, phenomenological ionic models aim to describe the collective effects of the ionic currents by a smaller number of state variables (usually 2–3) and parameters, and are obtained either by simplifying a complex ionic model [7], [16], or by trying to reproduce the shape of the action potential [1], [8]. In the field of personalised models, particular emphasis has been given to the models with two state variables, due to their numerical tractability and the reduced number of parameters to be constrained [4], [10], [20], [23], [27].

Among the available two state-variable ionic models, the one introduced by Mitchell and Schaeffer (MS) [16] is often employed in ventricular electro-physiology inverse problems [4], [21], [23]. This model is capable of reproducing the shape of the action potential and the restitution properties of the action potential duration (APD), [12], [14]. The MS model is characterised by 5 parameters. It is obtained by simplifying the model proposed by Fenton and Karma, [7] (3 state variables, 13 parameters), which in turn is obtained by simplifying the more complex Luo Rudy I [15] biophysical ionic model (8 state variables, 63 parameters).

**Fig. 1:**
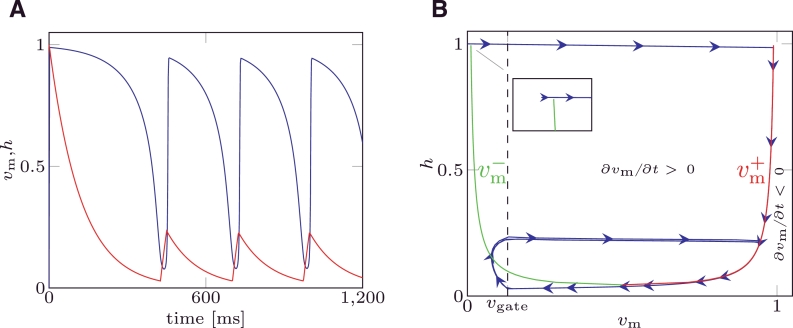
(A) State variable for MS model affected by pacemaker behaviour. (B) Nullclines (green: left branch, $v_{m}^{-}$; red: right branch, $v_{m}^{+}$) and phase portrait for MS model affected by pacemaker behaviour. Parameter values are reported in the Table 2 of example 2. (For interpretation of the references to colour in this figure legend, the reader is referred to the web version of this article.)

For some choices of the parameters, the MS model suffers from the so called “pacemaker cell behaviour”. That is, the trans-membrane potential cyclically depolarises and repolarises in the absence of any applied external stimulus, as depicted in Fig. 1A. In Fig. 1B (blue line) the solution of the MS model under pacemaker behaviour is depicted in the phase space. From an analysis of the MS model equations, it is possible to analytically define the relation between the gate variable and the trans-membrane potential delimiting the values of the state variables producing a depolarisation. This curve is called a nullcline, and it is split into a left branch, ($v_{m}^{-}$) and a right branch, ($v_{m}^{+}$) depicted in Fig. 1B. Once activated when the system moves towards the initial (at rest) condition, if the phase portrait crosses the nullcline branch $v_{m}^{-},$ the system “falls” into a condition where a depolarisation occurs, and a new action potential will be produced even though no external stimuli were applied. The sets of parameters that yield pacemaker cell behaviour do not delimit a closed region of the parameter space. This phenomenon has been observed and reported both in 0D and tissue models, [5], [22]. However, to the best of our knowledge there are no criteria for determining a priori which combination of parameters will produce pacemaker activity.

For patient specific modelling, this unwanted behaviour represents a problem for parameter estimation, since it necessitates a stability test for each estimated parameter set, [5].

Particularly affected by this phenomenon are the sequential data assimilation techniques [3], [9], [11]: since the values of the parameters are sequentially updated to minimise the discrepancy between the output of the model and the measurements of the system under study, if a combination of parameters yielding pacemaker behaviour is produced, the algorithm adopted could become unstable and diverge.

To overcome these limitations, in this paper we derive and introduce a two state-variable ionic model that describes the action potential phases by 5 parameters, has the same benefits as the MS model, and is robust to pacemaker behaviour. These characteristics make it suitable for generating personalised electrophysiology models for clinical applications, in particular when a sequential data assimilation technique is employed. This paper is organised as follows: in Section 2 we introduce the mathematical formulation of the new ionic model; in Section 3 we prove the absence of pacemaker behaviour in the phase plane; in Section 4 we derive an asymptotic derivation of the restitution curves and compare it to the one described in [16] for the standard MS model; and in Section 5 we compare the solutions and the restitution properties of the new model with the MS model for some numerical examples.

## 2 The modified Mitchell–Schaeffer ionic model

The standard MS ionic model [16] describes the ionic currents that flow across the cell membrane with a gated-inward ionic current, representing the current produced by the flux of the sodium ions, and an ungated outward ionic current, representing the current produced by the flux of the potassium ions. The two state variables characterising the MS model represent the electric potential of the cell membrane and the gate dynamics of the sodium ion channels. The model can be written in the generalised form proposed by [6]:

(1) $$\begin{array}{cc} \frac{\partial v_{m}}{\partial t} & =h\frac{\left(v_{m}+a\right)\left(v_{m}+a-\lambda \right)\left(1-v_{m}\right)}{\tau_{\mathrm{in}}}-\frac{v_{m}}{\tau_{\mathrm{out}}}+J_{\mathrm{stim}} \end{array}$$

(2) $$\begin{array}{cc} \frac{\partial h}{\partial t} & =\left\{ \begin{array}{cc} \frac{1-h}{\tau_{\mathrm{open}}} & v_{m}\leq v_{\mathrm{gate}}\\ -\frac{h}{\tau_{\mathrm{close}}} & v_{m}>v_{\mathrm{gate}} \end{array}\right. \end{array}$$

where *J*_stim_ is an externally applied electrical stimulus, *v*_m_ is the trans-membrane potential, *h* is the gate variable of the inward current, *v*_gate_ is the activation threshold potential and *τ*_in_, *τ*_out_, *τ*_open_, *τ*_close_ are the 4 time constants affecting the 4 characteristic phases of the trans-membrane potential. The standard MS model described in [16] is obtained by imposing a=λ=0; the parameters a and *λ* were introduced by [6] and used to control the excitability of the system.

The modified Mitchell–Schaeffer (mMS) ionic model presented in this paper is obtained by first replacing a=0 and $\lambda =v_{\mathrm{gate}}$ in (1): consequently, if *v*_m_ < *v*_gate_, then ∂*v*_m_/∂*t* < 0 and the system naturally evolves towards the rest condition instead of producing an action potential, in contrast to the original MS model where there is a range of values $v_{m}^{-}\left(h\right)\leq v_{m}<v_{\mathrm{gate}}$ yielding ∂*v*_m_/∂*t* > 0.

The effect of the potassium ion current is negligible when *v*_m_ ≤ *v*_gate_ and the cell is returning to a quiescent state. It is possible to introduce gating effects to the outward ionic current with the complement of the gate variable, (1−*h*), adopting an expression similar to the one introduced in [25]. This leads to the following system of ODEs:

(3) $$\begin{array}{cc} \frac{\partial v_{m}}{\partial t} & =h\frac{v_{m}\left(v_{m}-v_{\mathrm{gate}}\right)\left(1-v_{m}\right)}{\tau_{\mathrm{in}}}-\left(1-h\right)\frac{v_{m}}{\tau_{\mathrm{out}}}+J_{\mathrm{stim}} \end{array}$$

(4) $$\begin{array}{c} \frac{\partial h}{\partial t}=\left\{ \begin{array}{cc} \frac{1-h}{\tau_{\mathrm{open}}} & v_{m}\leq v_{\mathrm{gate}}\\ -\frac{h}{\tau_{\mathrm{close}}} & v_{m}>v_{\mathrm{gate}} \end{array}\right. \end{array}$$

Remark The robustness to pacemaker behaviour is obtained by modifying the cubic polynomial on the right hand side of Eq. (1). The addition of gating on the outward current has three advantages: first, when h=1 the threshold value of the transmembrane voltage above which an action potential is triggered is equal to *v*_gate_; second, for h=1 the transmembrane potential at the end of the upstroke will be $v_{m}=1$; third, as will be discussed in Section 4, the analytical solution of the mMS model coincides to the analytical solution of the MS model for the same set of ionic parameters for a particular choice of *v*_gate_.

## 3 Robustness to pacemaker behaviour

The study of the robustness of the mMS model to pacemaker behaviour consists in determining if the condition ∂*v*_m_/∂*t* > 0 could occur without an external stimulus being applied. In the phase plane, the nullclines of Eq. (1) for the MS model and Eq. (3) for the mMS model define the boundary between the region where the depolarisation occurs and the region where the system naturally evolves towards the rest condition; when *h* recovers towards its rest state h=1, if the phase portrait crosses the nullcline branch $v_{m}^{-},$ as depicted in Fig. 1B, then the system depolarises again and a new action potential is produced without an external stimulus having been applied.

The robustness of the mMS model to pacemaker behaviour is shown by proving that its phase portrait cannot intersect the nullcline branch $v_{m}^{-}$ unless an external stimulus is applied. To this aim, we first derive the nullcline of Eq. (3), we then determine the three characteristic points corresponding to the minimum and maximum potentials delimiting the region where depolarisation occurs, and the point where the $v_{m}^{-}$ and $v_{m}^{+}$ branches merge; we also compare these points with the corresponding points from the original MS model. Once the nullcline and characteristic points are known, we show that the phase portrait of mMS cannot cross the nullcline branch $v_{m}^{-}$ during the recovery of *h*.

### 3.1 Nullclines

Denoting the minimum value of *h* on the nullclines in the MS model by

(5) $$h_{\min}^{\mathrm{MS}}=\frac{4\tau_{\mathrm{in}}}{\tau_{\mathrm{out}}}$$

the nullclines of MS are defined in [16] as follows:

(6) $$\begin{array}{cc} v_{m} & =0\\ v_{m}^{\pm }\left(h\right) & =\frac{1}{2}\left(1\pm \sqrt{1-\frac{h_{\min}^{\mathrm{MS}}}{h}}\right) \end{array}$$

denoting by

(7) $$h_{\min}^{\mathrm{mMS}}={\left(1+\frac{\tau_{\mathrm{out}}}{4\tau_{\mathrm{in}}}{\left(1-v_{\mathrm{gate}}\right)}^{2}\right)}^{-1}$$

the minimum value of *h* on the nullcline of the mMS model, the nullclines of the new mMS model described in Eq. (3) are defined as follows:

(8) $$\begin{array}{cc} v_{m} & =0\\ v_{m}^{\pm } & \left(h\right)=\frac{1}{2}\left(1+v_{\mathrm{gate}}\pm \left(1-v_{\mathrm{gate}}\right)\sqrt{1-\frac{1-h}{h}\frac{h_{\min}^{\mathrm{mMS}}}{1-h_{\min}^{\mathrm{mMS}}}}\right) \end{array}$$

**Fig. 2:**
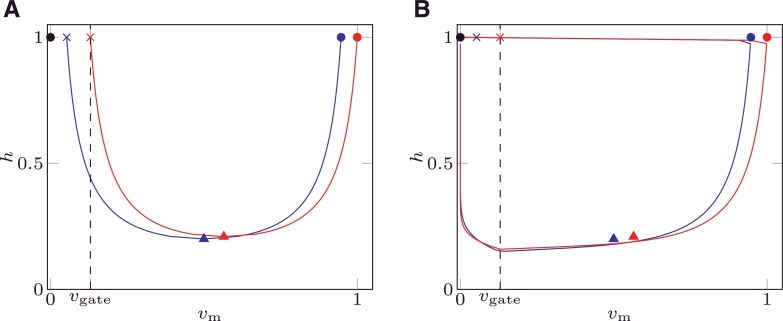
Nullclines (A) and phase portrait (B) of the mMS () and MS () models. The black dashed line represents $v_{m}=v_{\mathrm{gate}}$; the points () and () represent the points 1,2 and 3 for the mMS and MS models, respectively. The zeroth point (•) represents the rest (initial) state and coincides for both models. The values of the parameters were taken from [16].

Eqs. (6) for MS and (8) for mMS define two regions in the phase plane: the first one defined by the interval $v_{m}^{-}\left(h\right)<v_{m}<v_{m}^{+}\left(h\right)$ where ∂*v*_m_/∂*t* > 0; the second one defined by $v_{m}<v_{m}^{-}\left(h\right)$ and $v_{m}>v_{m}^{+}\left(h\right)$ where ∂*v*_m_/∂*t* < 0. Fig. 2A depicts the nullclines of MS () and mMS (). In the same figure, the black dashed line represents the threshold potential *v*_gate_ where *h* switches between closing and opening (recovery); the region *v*_m_ < *v*_gate_ of the phase plane is critical in the study of the robustness to pacemaker behaviour.

The following 4 characteristic points are defined for Eqs. (6) and (8) and are depicted in Fig. 2:

• **Point 0**: rest (initial) state. In both models, this point (•) is characterised by $\left(v_{m}=0,h=1\right)$ in the phase plane. This point does not belong to nullclines (6) and (8).
• **Point 1**: end of depolarisation from a rest state, $\left(v_{m}^{+}\left(1\right),h=1\right)$. This point characterises the value of *v*_m_ at the end of the depolarisation upstroke and it is equal to (1, 1) for mMS () and to $\left(\frac{1}{2}+\frac{1}{2}\sqrt{1-h_{\min}^{\mathrm{MS}}},1\right)$ () for MS.
• **Point 2**: merging point of the $v_{m}^{-}$ and $v_{m}^{+}$ branches. This point defines the minimum value of *h* on the nullcline: it approximates the state point where the phase portrait “falls off the nullcline”, and is equal to $\left(\frac{1+v_{\mathrm{gate}}}{2},h_{\min}^{\mathrm{mMS}}\right)$ () for mMS and to $\left(\frac{1}{2},h_{\min}^{\mathrm{MS}}\right)$ () for MS.
• **Point 3**: minimum potential on branch $v_{m}^{-}$$\left(v_{m}^{-}\left(1\right),h=1\right)$. This point represents the minimum value an external stimulus has to rise *v*_m_, to produce an action potential when the system is fully recovered. It is equal to (*v*_gate_, 1) () for mMS and to $\left(\frac{1}{2}-\frac{1}{2}\sqrt{1-h_{\min}^{\mathrm{MS}}},1\right)$ ( ) for MS.

In the phase plane, the phase portrait is defined as the curve described by the values (*v*_m_(*t*), *h*(*t*)) which constitute the solution of the ionic model. The phase portraits of MS () and mMS () are depicted in the right panel of Fig. 2 for a non-pacemaker solution; the phase portrait for a pacemaker solution for the MS model is depicted in Fig. 2B.

### 3.2 Robustness

The nullcline and characteristic points were defined above. From these points we show that the phase portrait of mMS cannot cross the nullcline branch $v_{m}^{-}$ during the recovery of *h* unless an external stimulus is applied. To simplify the notation, we denote the value of the trans-membrane potential of Point 2 in mMS by

$$\begin{array}{cc} v_{m}^{*} & =v_{m}\left(h_{\min}^{\mathrm{mMS}}\right)=\frac{1+v_{\mathrm{gate}}}{2} \end{array}$$

We then split the phase portrait into two parts: the repolarisation, defined by the interval $v_{\mathrm{gate}}\leq v_{m}\leq v_{m}^{*}$ for mMS and the interval *v*_gate_ ≤ *v*_m_ ≤ 1/2 for MS, and the recovery, when *h* returns to its initial value, defined by *v*_m_ < *v*_gate_.

For both models, during the repolarisation *v*_m_ approaches *v*_gate_ at a rate proportional to 1/*τ*_out_. Since *v*_m_ ≥ *v*_gate_, *h* decreases at a rate proportional to 1/*τ*_close_ and the phase portrait moves away from the nullcline branch $v_{m}^{-},$ lying in the region of the phase plane where no depolarisation can occur.

When *v*_m_ < *v*_gate_, *h* recovers at a rate proportional to 1/*τ*_open_, while the time derivative of *v*_m_ is still negative, as depicted in Fig. 2B. The system moves towards Point 0. Since $v_{m}^{-}\left(h=1\right)=v_{\mathrm{gate}}$ in mMS, the phase portrait will never cross the nullcline branch $v_{m}^{-},$ independently of the value of *τ*_open_. Unless an external stimulus is applied, the system evolves towards Point 0, and no pacemaker behaviour will occur. In contrast, the MS model can cross the nullcline branch $v_{m}^{-}$ if *τ*_open_ is small enough, since there is a non-empty region where $v_{m}^{-}\left(1\right)<v_{m}\leq v_{\mathrm{gate}}$.

We tested the robustness of the MS and the mMS models to pacemaker behaviour on a regular grid set of 57,800 parameters chosen with the range and the spacing reported in Table 1. The set of parameters characterised by $h_{\min}^{\mathrm{MS}}\geq 1$ were excluded since this choice does not produce an action potential. The statistics of the pacemaker behaviour was thus evaluated on a total of 52598 samples. For both models, an external stimulus was applied at t=0 ms and the numerical solution was evaluated over 1200 ms; parameter sets presenting more than one depolarisation were considered pacemaker. For 3042 combinations (≃6% of the cases) MS displayed pacemaker behaviour, while the mMS was robust in all of the 52,598 tests.

## 4 Asymptotic derivation of the restitution curve

Following the same procedure adopted in [16], it is possible to derive an explicit leading order asymptotic approximation for the APD restitution curve of mMS, based on the assumption

(9) $$\tau_{\mathrm{in}}\ll \tau_{\mathrm{out}}\ll \tau_{\mathrm{open}},\tau_{\mathrm{close}}$$

From assumption (9) it follows that the time constants of Eq. (3) are infinitesimal if compared to the time constants of Eq. (4) and thus the duration of the depolarisation and repolarisation phases can be neglected when compared to the APD and recovery durations.

### 4.1 Single stimulus: maximum APD (APD_max_)

In this paper we adopt the same definition introduced in [16] for APD as the elapsed time during which *v*_m_ ≥ *v*_gate_.

Due to the separation of the time scale introduced by assumption (9) and considering a cell membrane initially in the rest state (h=1,$v_{m}=0$), then the action potential generated by an externally applied stimulus is decomposed into the following 4 phases:

1. **Depolarisation:** when the external stimulus is applied with sufficient duration *T*_stim_ and intensity such that *v*_m_(*T*_stim_) > *v*_gate_, the fast inward current dominates and the potential rises quickly to (h=1,$v_{m}=1$) on the right nullcline with a characteristic time proportional to *τ*_in_.
2. **APD:** when the cell membrane is fully depolarised and the gate progressively closes, the inward and the outward ionic currents balance each other. The system evolves following the right nullcline on a time scale proportional to *τ*_close_.
3. **Repolarisation:** when $\left(h,\;v_{m}\right)\;∼\;\left(h_{\min}^{\mathrm{mMS}},\;v_{m}^{*}\right),$ the solution falls off the nullcline, the outward current dominates, and the potential drops toward $v_{m}=0$ on a time scale proportional to *τ*_out_.
4. **Recovery:** when $v_{m}=v_{\mathrm{gate}},$ the gate variable slowly re-opens and recovers with a characteristic time scale of *τ*_open_. Since the fast outward current, with characteristic time scale of *τ*_in_, dominates, the potential drops to $v_{m}=0$ rapidly, due to assumption (9).

The durations of the depolarisation and repolarisation are negligible if compared to the APD and recovery duration: thus, at leading order, APD can be approximated as the time elapsed to evolve from h=1 to $h=h_{\min}^{\mathrm{mMS}},$ obtained from (4) as follows:

(10) $${\mathrm{APD}}_{\max}=\tau_{\mathrm{close}}\ln\left(\frac{1}{h_{\min}^{\mathrm{mMS}}}\right)$$

### 4.2 Multiple stimuli: restitution curve

In this section we derive a leading order approximation of the APD when two (or more) stimuli are applied. The first stimulus is applied at t=0ms to a fully recovered tissue; the second stimulus is applied at time t=Sms, to a system not fully recovered.

Denoting by APD_1_ the APD following the first stimulus and by DI = S-APD_1_ the diastolic interval (DI), that is the time elapsed between the end of the APD and the second pacing stimulus, from Eq. (4) there follows

(11) $$h\left(S\right)=1-\left(1-h_{\min}^{\mathrm{mMS}}\right)e^{-\frac{\mathrm{DI}}{\tau_{\mathrm{open}}}}$$

Due to assumption (9), the depolarisation duration is negligible if compared to APD and recovery duration; thus, from (4) and the initial condition (11), the following relation between APD and DI, called restitution, holds:

(12) $${\mathrm{APD}}_{n+1}=\tau_{\mathrm{close}}\ln\left(\frac{1-\left(1-h_{\min}^{\mathrm{mMS}}\right)e^{-\frac{{\mathrm{DI}}_{n}}{\tau_{\mathrm{open}}}}}{h_{\min}^{\mathrm{mMS}}}\right)$$

**Fig. 3:**
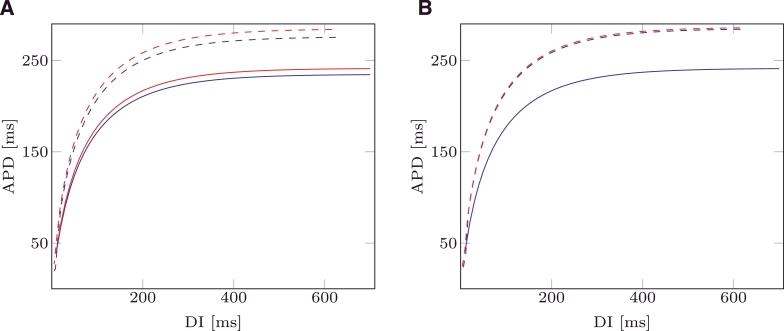
Analytic APD restitution curves for mMS () and MS () models. Evaluated APD restitution curves for mMS () and MS () models. The values of the ionic parameters were taken from [16], and are $\tau_{\mathrm{in}}=0.3,$$\tau_{\mathrm{out}}=6,$$\tau_{\mathrm{open}}=120,$$\tau_{\mathrm{close}}=150$. (A) $v_{\mathrm{gate}}=0.13$; (B) $v_{\mathrm{gate}}=v_{\mathrm{gate}}^{*}$.

In Fig. 3A the analytical APD restitutions are depicted for mMS () and MS () and $v_{\mathrm{gate}}=0.13$. In the same figure dashed lines depict the APD restitution computed by numerically solving MS and mMS. In Fig. 3B, the same restitutions are plotted for $v_{\mathrm{gate}}=v_{\mathrm{gate}}^{*}=1-\sqrt{1-h_{\min}^{\mathrm{MS}}}$: this value is evaluated by imposing $h_{\min}^{\mathrm{mMS}}=h_{\min}^{\mathrm{MS}}$ and produces identical analytical restitution curves for both models. This choice of the activation threshold also produced similar computed restitution curves for the two models (dashed lines, Fig. 3B).

The restitution curves were computed by applying an $s_{1}\_s_{2}$ protocol [17]. Briefly, the model is repetitively stimulated with an inter-pacing interval *s*_1_ until a steady state is achieved, (in the present paper, $s_{1}=1000\;\mathrm{ms},$ applied 100 times to achieve a limit cycle), and then a single stimulus is applied at an interval *s*_2_ < *s*_1_ and the system is left to evolve. This procedure is applied for different values of *s*_2_ and the APD is measured after the premature stimulus is applied. In the present paper, the values of the premature stimulus *s*_2_ < *s*_1_ were chosen with the following sequence:

$$\begin{array}{cc} & s_{1}^{0}=900\;\mathrm{ms}\\ & s_{1}^{i+1}=0.98s_{1}^{i}\;s_{1}^{i+1}\geq 200\;\mathrm{ms} \end{array}$$

Remark Expressions (10) and (12) are formally equivalent to those obtained in [16] for MS and differ only in the expression of Point 2 defined in Section 3.1. While in the MS model this point is defined by (5) and does not depend on *v*_gate_, in the mMS model this point is defined by expression (7) which introduces a dependence on *v*_gate_ in expressions (10) and (12).

Since the APD is defined as the elapsed time during which *v*_m_ ≥ *v*_gate_, expressions (10) and (12) are able to account for this dependency. As a demonstration, if $v_{\mathrm{gate}}=1.0$ is chosen, one expects no APD will be generated, independently of the choice of the other 4 ionic parameters. The analytic restitution expression of the MS model still will furnish a non-zero APD in this case. In contrast, from expressions (10) and (11) the restitution equation for the mMS model predicts an APD of 0 ms.

## 5 Numerical examples

To compare the functional characteristics of the MS and mMS models we compare model simulations for a stable action potential, an action potential affected by pacemaker behaviour, a dynamic restitution protocol and pacemaker behaviour in a tissue simulation.

Time discretisation was performed by a backward Euler method and non-linearities were treated by Newton iterations.

A time step dt=0.005ms was chosen; to test the accuracy of the chosen time step we evaluated the numerical solution for the set of parameters reported in [16] with a time step dt=0.005ms and a time step dt=0.0005ms. The maximum and the *L*^2^ differences between the solutions were equal to 0.004 and 0.005 respectively, (i.e., ≃ 0.5% of the maximum value of *v*_m_). The externally applied stimulus is characterised by an intensity of $1.0\;{\mathrm{ms}}^{-1}$ and a duration of 0.4 ms.

In Section 5.1 we deal with the ionic parameters taken from [16] that do not provide any pacemaker activity for MS.

In Section 5.2 we adopt the parameters reported in Table 2 that yield pacemaker behaviour for the MS model. We will show that while the MS model will furnish a pacemaker solution, the mMS model will be robust to this behaviour.

In Section 5.3 we evaluate the dynamic restitution curves for both models with the parameter set defined in [16] and we show that the differences between the two models in the dynamic APD restitutions are small.

In Section 5.4 we consider a homogeneous tissue slab characterised by the parameters reported in Table 3, stimulated by a cross-field protocol. This set of parameters did not produce any pacemaker behaviour for the MS model in a single cell, nor in a 1D tissue string for the same parameter set and model conditions, but did show pacemaker behaviour in two dimensions. We show that the mMS model is robust to pacemaker behaviour under these conditions.

### 5.1 Example 1: pacemaker free MS

**Fig. 4:**
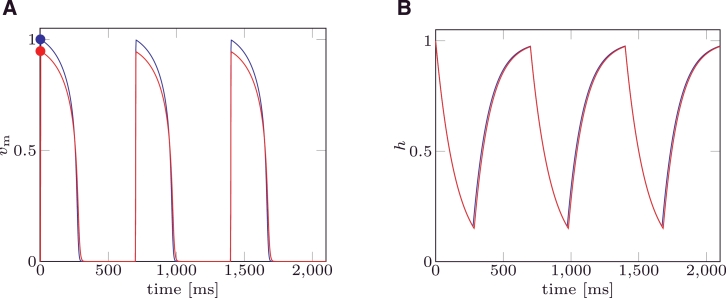
(A) Trans-membrane potential (*v*_m_) for mMS () and MS () models. Circles () represent the values ($v_{m}^{+}\left(1\right)$) characterising Point 1 for mMS and MS, respectively. (B) Gate variable for mMS () and MS () models. Parameter values were taken from [16], and are $\tau_{\mathrm{in}}=0.3,$$\tau_{\mathrm{out}}=6,$$\tau_{\mathrm{open}}=120,$$\tau_{\mathrm{close}}=150,$$v_{\mathrm{gate}}=0.13$.

In the first example the MS and the mMS models were solved with the parameter set taken from [16]. This choice yields a pacemaker free MS model. Three stimuli were applied at an inter-pacing interval T=700ms. For both models the trans-membrane potential (Fig. 4A) and the gate variable (Fig. 4B) functions of time are depicted.

The dynamics of the gate variable *h* do not significantly differ between the two models, while the trans-membrane potential *v*_m_ obtained by solving the mMS model only differs in the maximum value reached at the end of the depolarisation.

**Fig. 5:**
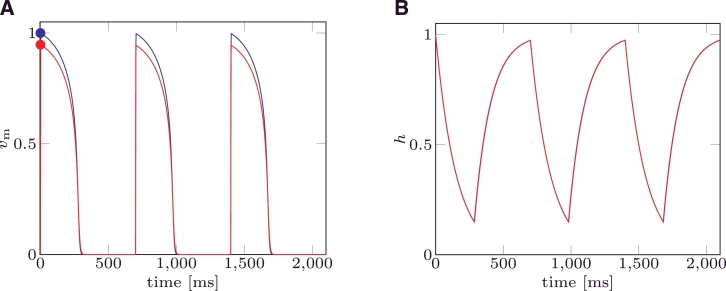
(A) Trans-membrane potential (*v*_m_) for mMS () and MS () models. Circles () represent values ($v_{m}^{+}\left(1\right)$) characterising Point 1 for mMS and MS, respectively. (B) Gate variable for mMS () and MS () models. Values for the ionic parameters were taken from [16], and are $\tau_{\mathrm{in}}=0.3,$$\tau_{\mathrm{out}}=6,$$\tau_{\mathrm{open}}=120,$$\tau_{\mathrm{close}}=150,$ while $v_{\mathrm{gate}}=v_{\mathrm{gate}}^{*}$.

In Fig. 5 the trans-membrane potential *v*_m_ and the gate variable *h* are plotted when a value of $v_{\mathrm{gate}}=v_{\mathrm{gate}}^{*}$ is chosen. This choice of *v*_gate_ yields the same analytical restitution curves for the mMS and the MS models.

### 5.2 Example 2: MS with pacemaker activity

**Fig. 6:**
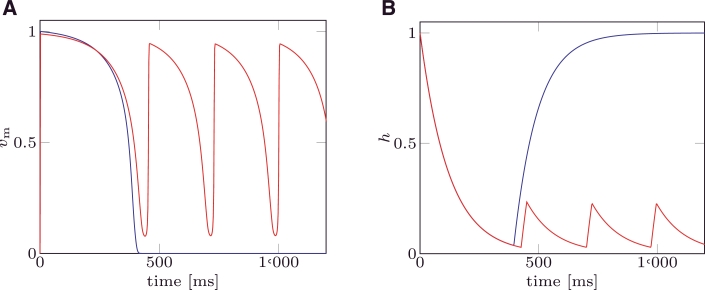
(A) Trans-membrane potential (*v*_m_) for mMS () and MS () models. (B) Gate variable (*h*) for mMS () and MS () models. Parameter values are reported in Table 2.

In the second example we characterise both models with the parameter set summarised in Table 2. This choice leads to a pacemaker solution in the MS model. The system is paced once at t=0 and the solution is evaluated until t=1200ms. The results are plotted in Fig. 6; while the mMS model presents only one action potential, the MS cyclically depolarises (with 4 activations during the simulation period).

**Fig. 7:**
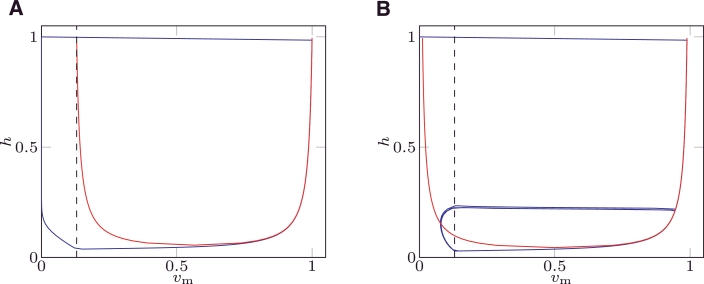
Phase portrait () and nullclines () for the modified MS model (A) and the standard MS model (B). Black dashed line ($v_{m}=v_{\mathrm{gate}}$) represents the threshold where *h* switches to recovering. Parameter values reported in Table 2 were taken.

In Fig. 7 the nullclines and the phase portrait are depicted for both models. The mMS model (Fig. 7A) recovers without crossing the left nullcline, since the recovery of *h* is far from the nullcline. The MS model (Fig. 7B) crosses the left nullcline during recovery and thus re-depolarises entering into a periodic limit cycle.

### 5.3 Example 3: dynamic restitution curves

Dynamic restitution curves are used to characterise the value of the APD when the tissue is periodically paced with a pacing period *S*. As discussed in [16], at larger values of *S*, each stimulus produces an action potential, yielding a 1:1 correspondence between *S* and APD, called 1:1 behaviour. As *S* decreases, this 1:1 behaviour eventually becomes unstable; as a result, two possible behaviours can be presented:

• A 2:1 behaviour: only every other stimulus produces an action potential;
• A 2:2 behaviour (alternans): each stimulus will produce an action potential, but the APD periodically alternates between short and long.

For both models, dynamic restitution curves were numerically evaluated. For each pacing period *S*, the system was periodically paced by applying 104 external stimuli and the APD was computed for each of the last 4 applied stimuli. When the APD values of the last 4 stimuli all coincide, the behaviour was considered of type 1:1. If the APD presented two different alternating values and all of the 4 stimuli produced an action potential the behaviour was considered of type 2:2 (alternans). Lastly, if the APD presented only one value and the 4 stimuli produced only two activations, the behaviour was considered to be of type 2:1. The examples tested did not present any type 2:1 behaviour.

Dynamic restitutions were evaluated for the parameters of Table 2 and for $v_{\mathrm{gate}}=v_{\mathrm{gate}}^{*}$ as defined in Section 4.2. The pacing interval *S* was decremented from 700 ms to 300 ms with a step of 100 ms and then from 280 ms with a step of 2 ms to the first value that did not produce APD. For $v_{\mathrm{gate}}=0.13$ no APD were produced for *S* = 256 ms for the mMS model and *S* = 266 ms for the MS model; the dynamic restitution of the MS model bifurcates at *S* = 278 ms with two APD values differing by 10 ms, while the mMS model bifurcates at 270 ms with two APD values differing by 230 ms. For $v_{\mathrm{gate}}=v_{\mathrm{gate}}^{*}$ no APD was produced for *S* = 266 ms for the mMS model and *S* = 268 ms for the MS model, while bifurcations occurred at *S* = 280 ms with two APD values differing by 10 ms in the MS model, and at *S* = 278 ms with two APD values differing by 240 ms in the MS model. The pacing periods where either APD bifurcates or no APD was produced differ by 2 ms between both models, a difference comparable with the step used to decrement *S*.

**Fig. 8:**
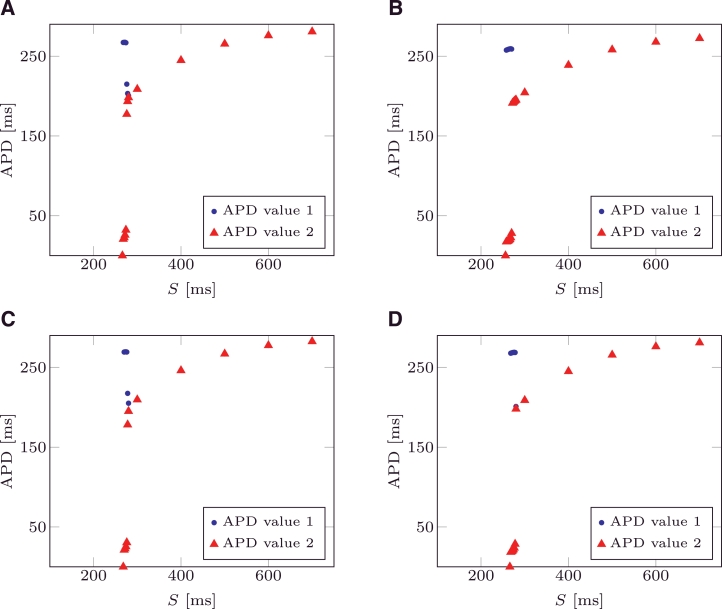
(A) Dynamic APD restitution for MS model, $v_{\mathrm{gate}}=0.13$. (B) Dynamic APD restitution for the mMS model, $v_{\mathrm{gate}}=0.13$. For two subsequent pacings, APD value are depicted as () and (). When circles and triangles are no longer superimposed, bifurcation occurs. (C) Dynamic APD restitution for MS model, $v_{\mathrm{gate}}=v_{\mathrm{gate}}^{*}$. (D) Dynamic APD restitution for the mMS model, $v_{\mathrm{gate}}=v_{\mathrm{gate}}^{*}$. (For interpretation of the references to colour in the text, the reader is referred to the web version of this article.)

In Fig. 8A the dynamic restitution of the MS model is depicted, while in Fig. 8B the same curve is depicted for the mMS model. In Fig. 8C and D the dynamic restitutions are depicted for the mMS and the MS models respectively. The two different APD values are depicted with a blue circle and a red triangle (second stimulus). When the circles and triangles are no longer superimposed, bifurcation occurs.

### 5.4 Example 4: 2D model with a cross-field stimulus

In this example we consider an homogeneous isotropic tissue slab measuring 5 cm × 5 cm; the tissue electrophysiology is described by the mono-domain model [13] and solved with the CARP [18], [26] finite element code. The equations were discretised in space with a triangular mesh with a characteristic size h=189μm and in time with a time step of 0.1 ms. The model was simulated for t=3500ms.

The tissue electrophysiology was characterised by the parameters reported in Table 3. These parameters did not generate any pacemaker activity in simulations of an isolated single cell or in a 1D domain.

The tissue was stimulated by the cross field stimulation protocol, [19]. Briefly, an external stimulus was applied to two orthogonal regions at two different stimulation times to trigger a spiral activation pattern. In this work the first stimulus *s*_1_ was applied at t=0ms on the left edge of the slab between x=0 and x= 0.2 cm, and *s*_2_ was applied at t=290ms on the bottom edge of the slab between y=0 and y= 0.25 cm. For both stimuli, a current intensity of $J_{\mathrm{stim}}=$$2.0\;{\mathrm{ms}}^{-1}$ with a duration of $T_{\mathrm{stim}}=$ 0.6 ms were employed.

**Fig. 9:**
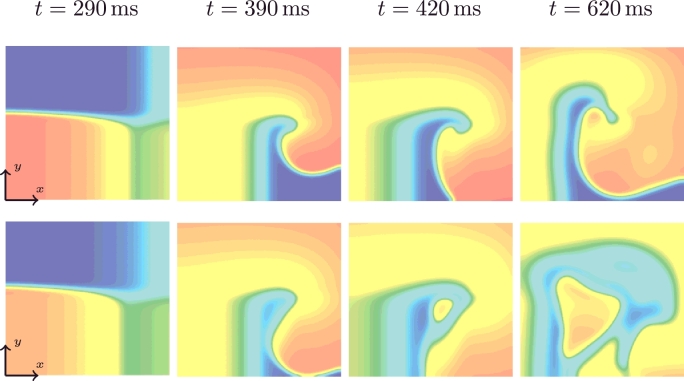
Trans-membrane potential followed by a cross-field stimulus for the mMS (top) and the MS (bottom) models at times *t* = 290, 390, 420 and 620 ms.

The transmembrane potentials of the mMS (top row) and the MS (bottom row) models are depicted in Fig. 9 for t=290ms (*s*_2_ is applied), t=390ms (a spiral wave is generated, no pacemaker activity is present), t=420ms (pacemaker behaviour appears in the MS model) and t=620ms (presence of pacemaker behaviour in the MS model). A video of the whole simulation can be found in the online supplement.

The mMS model generated a spiral re-entry wave that broke up at t=2080ms and terminated at t=2470ms, whereas the MS model showed a pacemaker behaviour, initiated by a pacemaker beat at t=420ms (Fig. 9, third column) and which persisted over the entire simulation period.

## 6 Discussion and conclusions

In this paper we introduced a novel two state-variable ionic model, obtained by modifying the standard Mitchell–Schaeffer (MS) ionic model. The new model has the same benefits as the standard MS model and is proven to be robust to pacemaker behaviour. Previously the MS model had been adapted to introduce pacemaker behaviour [6]. The nonlinear term characterising the inward ionic current was modified by introducing two new parameters. In this paper, we presented a model that is robust to pacemaker behaviour by modifying the inward current in a similar manner. Unlike [6], in this paper we also gated the outward current. This yields three advantages: first, the threshold value of the transmembrane voltage above which an action potential is triggered corresponds to *v*_gate_; second, at the end of the depolarisation, $v_{m}=1$; third, the analytical solution of the mMS model coincides with the analytical solution of the MS model for the same set of ionic parameters and $v_{\mathrm{gate}}=v_{\mathrm{gate}}^{*}$.

We then introduced an asymptotic derivation of the restitution curves, obtaining a relation formally equivalent to that obtained in [16]. We also showed, through numerical examples, that there are only small differences in the action potential introduced by the modified MS (mMS) equations compared to the original MS model when the same set of parameters are adopted. We also compared the APD dynamic restitution curves obtained by numerically solving both models; we showed that the mMS is able to reproduce the APD alternans and that these occur under a similar pacing regime in both models. Last, we demonstrated the robustness of the new model even when incorporated into a mono-domain tissue simulation, confirming its applicability in tissue scale patient-specific modelling.

## Figures and Tables

**Table 1:** Parameter set ranges and step adopted for testing the robustness to pacemaker behaviour of the mMS model. For the same set of parameters the behaviour of MS was also evaluated.

	*τ*_in_ [ms]	*τ*_out_ [ms]	*τ*_open_ [ms]	*τ*_close_ [ms]
min	0.05	0.5	60	60
max	0.5	10	220	220
step	0.05	0.5	10	10

**Table 2:** Parameter values used for testing pacemaker stability.

*v*_gate_	*τ*_in_ [ms]	*τ*_out_ [ms]	*τ*_open_ [ms]	*τ*_close_ [ms]
0.13	0.1	9.0	100	120

**Table 3:** Parameter values used for testing pacemaker stability.

*v*_gate_	*τ*_in_ [ms]	*τ*_out_ [ms]	*τ*_open_ [ms]	*τ*_close_ [ms]	conductivity [cm^2^/s]
0.13	0.15	6.5	90	85	1.75

## Appendix A Supplementary materials

Supplementary Data S1 Supplementary Raw Research Data. This is open data under the CC BY license http://creativecommons.org/licenses/by/4.0/ Supplementary Data S1

Supplementary Data S2 Supplementary Raw Research Data. This is open data under the CC BY license http://creativecommons.org/licenses/by/4.0/ Supplementary Data S2
